# Funding evidence‐based conservation

**DOI:** 10.1111/cobi.13991

**Published:** 2022-10-10

**Authors:** Danni Parks, Nida Al‐Fulaij, Clare Brook, Stuart H. M. Butchart, Jean‐Gaël Collomb, David Cope, Simon Dowell, Becky Falkingham, Winifred F. Frick, Douglas Gibbs, Emily E. Gray, Nicolas Heard, Anastasios (Tasso) Leventis, Kate Mastro, Helen Meredith, Simon Mickleburgh, Florence Miller, Matthew Muir, Rascha J. M. Nuijten, Nancy Ockendon, Nisha R. Owen, Jacob R. Owens, Jon Paul Rodríguez, Sugoto Roy, Elizabeth Tully, Jean‐Christophe Vié

**Affiliations:** ^1^ The Whitley Fund for Nature London UK; ^2^ People's Trust for Endangered Species London UK; ^3^ Blue Marine Foundation London UK; ^4^ BirdLife International Cambridge UK; ^5^ Wildlife Conservation Network San Francisco California USA; ^6^ John Spedan Lewis Foundation London UK; ^7^ North of England Zoological Society Chester UK; ^8^ The National Trust Swindon UK; ^9^ Bat Conservation International Washington DC USA; ^10^ Darwin Initiative, Department for Environment, Food and Rural Affairs London UK; ^11^ Woodland Trust Grantham UK; ^12^ Mohamed bin Zayed Species Conservation Fund Abu Dhabi United Arab Emirates; ^13^ A.P. Leventis, The A. G. Leventis Foundation London UK; ^14^ The Wildlife Conservation Society Bronx New York USA; ^15^ Amphibian Survival Alliance London UK; ^16^ The Rufford Foundation London UK; ^17^ Environmental Funders Network Buckinghamshire UK; ^18^ United States Fish and Wildlife Service International Affairs Falls Church Virginia USA; ^19^ Future for Nature Foundation Arnhem The Netherlands; ^20^ Endangered Landscapes Programme Cambridge Conservation Initiative Cambridge UK; ^21^ On the EDGE Conservation London UK; ^22^ Los Angeles Zoo & Botanical Gardens Los Angeles California USA; ^23^ IUCN Species Survival Commission Venezuelan Institute for Scientific Investigation and Provita Caracas Venezuela; ^24^ IUCN SSC Cat Specialist Group Ittigen Switzerland; ^25^ WCS Climate Adaptation Fund Bronx New York USA; ^26^ Fondation Franklinia Genève Switzerland

The magnitude of the biodiversity crisis is widely accepted, as is the need for substantive action implementing the most effective interventions in the right locations (IPBES, 2019). Funding is a key driver of conservation work: its availability and funder preferences often determine what can be done. As representatives of 25 organizations that support conservation projects, we take seriously our responsibilities to ensure that funding decisions support evidence‐based conservation actions.

There is a growing understanding of the importance of considering the evidence for the effectiveness of actions in decision‐making and the risks of not doing so (Brest & Harvey, 2018). However, at present, evidence is not routinely considered when planning and designing conservation projects, despite the fact that some actions are more effective than others and some routine or common sense actions have been proved not to be effective at all (Sutherland & Wordley, 2018). For example, a recent comparison of the cost‐effectiveness of various actions to conserve orangutans (*Pongo* spp.) found that some (habitat protection and patrolling) were more than twice as effective as others (rescue and rehabilitation or habitat restoration) (Santika et al., 2022). Therefore, we believe that there is an opportunity for funders to catalyze a step change in evidence use in conservation planning. Asking applicants for funding to assess the evidence for what has worked––and what has not––when designing projects can help identify approaches with a higher likelihood of success, leading to increases in efficacy and improved outcomes. This is supported by a study that showed that asking conservation practitioners to consider the evidence base can change their decisions (Walsh et al., 2015). In addition, both the volume (Appendix S1) and accessibility (Piwowar et al., 2018) of evidence for the effectiveness of a wide range of conservation actions are growing rapidly, enhancing the practicality and likely impact of such a requirement.

Our organizations promote consideration of the evidence at a pertinent stage in the proposal‐development process, and we compiled 10 practical approaches for doing so (Table 1). Whichever approach is used, it is important that organizations review and incorporate new evidence (whether generated by their own project or others) at appropriate, regular intervals. Appendix S1 contains guidance for funders and applicants to demonstrate that key decisions in proposals are evidence‐based. We stress that we encourage continued innovation and the testing of practices to generate new evidence and that reporting unsuccessful interventions is as important as documenting success.

**TABLE 1:** Ten approaches for ensuring conservation grant applicants reflect on evidence

Approach	Description	Example of adopting organizations
Application form contains a question about evidence	A section in the application form asks about evidence for the effectiveness of any proposed conservation actions. This is a straightforward and effective approach. However, it may be seen as too bureaucratic, time‐consuming, or challenging. It may also not be appropriate if most applications do not involve conservation actions or if decision‐making takes place at a different stage of project development.	The Whitley Fund for Nature, People's Trust for Endangered Species, Birdlife International, John Spedan Lewis Foundation, North of England Zoological Society, The National Trust, Bat Conservation International, Woodland Trust, Mohamed bin Zayed Species Conservation Fund, The Rufford Foundation, and WCS Climate Adaptation Fund
Applicants asked to describe their evidence use somewhere in the proposal	This approach is less formal than having a specific question about evidence in the form. Perhaps more appropriate for smaller grants or applicants less used to applying for funding.	People's Trust for Endangered Species, Amphibian Survival Alliance, and Future for Nature Foundation
Second application stage asks about evidence	Some funders have an initial short application form, followed by a second stage, in which a subset of applicants provide further details. Funders can ask for evidence of effectiveness of proposed actions in the second stage, where relevant. This can reduce workload in the first stage and permits a general application process for a wide range of projects, with only practical conservation proposals required to provide evidence in the second stage.	Endangered Landscapes Programme
Applicants are asked to justify assumptions underpinning their theory of change	Many applications request a theory of change elucidating how the proposed actions are likely to result in the desired outcomes. Funders can ask for the evidence base for the assumptions underlying the theory of change.	Endangered Landscapes Programme
Grantees are asked to describe evidence use as part of reporting	The grant application and contract state the expectation that decision‐making processes will be evidence based. Grantees’ reports to the funder then describe how evidence was used in decision‐making and why key actions were chosen. This is appropriate where key decisions are made during the project, rather than before submitting the application.	
Funders check evidence themselves	If assessing evidence is considered too onerous or off‐putting for applicants, funders can themselves check the evidence for proposed actions during the selection process.	Future for Nature Foundation and the U.S. Fish and Wildlife Service International Affairs
Evidence is considered during project codevelopment	Some funders may not use a straightforward application process but instead codesign projects with potential grantees. The funder may then identify the evidence and discuss how to use this in project planning with potential grantees.	On the EDGE Conservation
Process for using evidence is described	Applicants are asked to describe the process by which relevant evidence will be identified and considered during their project. This may be appropriate for complicated projects requiring numerous decisions or sets of projects (or even an organization) that are funded before details of activities to be undertaken are known.	
Evidence use is clearly stated in selection criteria	A transparent criterion makes it clear that only proposals that use evidence‐based decision‐making will be considered for funding.	Woodland Trust and WCS Climate Adaptation Fund
Monitoring and enhancing index of evidence use	Funders monitor the level of evidence use (e.g., percentage of projects or funding supported by evidence) and set targets for annual improvement.	U.S. Fish and Wildlife Service International Affairs

We hope others will adopt this approach so that consideration of the relevant evidence becomes a routine part of decision‐making in conservation and beyond, resulting in enhanced effective practice on the ground.

## Supporting information

Evidence‐based funding applications: a guide for conservation funders and applicants
