# Supplementary material for: Funding evidence‐based conservation
Source: Conserv Biol. 2022 Oct 10;36(6):e13991. doi: 10.1111/cobi.13991 (PMC10092027; doi:10.1111/cobi.13991)
Supplement: Supplementary file 1 — Evidence‐based funding applications: a guide for conservation funders and applicants [file COBI-36-0-s001.docx]

**Appendix S1. Evidence-based funding applications: a guide for conservation funders and applicants**

Funders are very welcome to adapt this material to suit their own processes and to use as guidance for applicants

**Purpose of the document**

There is growing awareness of the importance of using evidence in decision making in conservation. The development and writing of funding applications is often a key point for decision making in project planning. Therefore, it is potentially beneficial to both funders and applicants if funding application processes provide an opportunity for applicants to describe the evidence underpinning the actions they propose.

However, the most effective way to present the evidence base, enabling the applicant to demonstrate the rigour of their decision-making process and the funder to assess this, is not always obvious. It is not realistic to expect applicants to provide a full description of the identification, collation and evaluation of evidence for each of the numerous decisions that are likely to be part of any conservation project. Instead, it is more useful to ask applicants to describe the evidence base for the most important decisions within a proposal, with a particular focus on those that may have substantial or sensitive consequences. In doing this, applicants also demonstrate that they can and do check the evidence as part of their planning processes, increasing funders’ confidence in their wider decision making.

It is important to note that the purpose of this guidance is to ensure that applicants have checked the available evidence when planning their conservation interventions. An often-raised concern is that this may stifle innovative practice. However, most funders look favourably on innovations that have been carefully thought through, and stating that a proposed intervention has little or no existing relevant evidence may still be perfectly acceptable, so long as the novelty of the planned action has first been confirmed. It is those actions that evidence suggests may be ineffective or even harmful that this process aims to screen out. If a novel, untested action is included in a proposal, it is particularly important that the applicant is encouraged to gather new evidence to understand its effects.

This document provides guidance that funders can use when asking applicants to describe the evidence for a set of key decisions in their funding applications. It aims to enable applicants to demonstrate an appropriate level of evidence consultation has taken place and allow funders to assess this consistently across proposals, without being overly onerous for either group.

**The Guidance**

***1. What is evidence***

The evidence base for a conservation decision may come from published literature (books or journals), manuals and reports (grey literature), previous personal experience, or be received through word of mouth from colleagues, peers or expert advisors. If available, peer-reviewed scientific research is often most informative for evaluating actions’ impacts on species, habitats or ecosystems, or assessing the generality of the effects of conservation actions. Local knowledge and experience are usually critical in providing information on local context and previous responses.

To identify the scientific evidence describing the likely outcomes of conservation actions, we recommend starting with a search of the [Conservation Evidence](https://www.conservationevidence.com/) database, which provides reviews of over 3,500 actions comprehensively covering many species groups and habitats. Other useful sources of evidence include: [CEEDER](https://environmentalevidence.org/ceeder/), a library of reviews on a range of topics; [Applied Ecological Resources](https://www.britishecologicalsociety.org/applied-ecology-resources/), a searchable database of journal articles; the [IUCN Red List of Threatened Species](https://www.iucnredlist.org/), containing assessments of species’ extinction risk based on data on [species’](https://www.iucnredlist.org/) population size and trends, distribution, threats and conservation actions, and the [IUCN Red List of Ecosystems](https://assessments.iucnrle.org/) containing assessments of ecosystems’ risk of collapse.

The two key characteristics of a piece of evidence are its reliability and its relevance. The reliability (or quality) give an indication of whether the same results would be replicated if the study was repeated. This will be determined by the way in which the data were collected (e.g. were there control sites for the treatment or intervention, were actions replicated, was randomisation used to reduce the likelihood of confounding factors?). The relevance refers to how similar the context where the results were collected is to the context where the proposed project is taking place (e.g. Are the species, habitat, soil, altitude or climate similar in the evidence cited and the proposed project? Are the two areas geographically close?). Such factors will affect whether a similar response can be expected in the proposed project. Both these components need consideration when assessing how much weight to put on a particular piece of evidence in the decision-making process.

***2. Which decisions?***

Not all the decisions taken during the development of a conservation project can or should be fully evidence-checked. It may not be important to provide evidence for minor interventions or those where the outcome is clearly beneficial (for example a decision to build a barrier to stop dirt bikes accessing a protected area with vegetation that is vulnerable to disturbance). The key decisions, where it is important for both funders and applicants that a thorough and robust evidence search and evaluation has taken place, are those where there is potentially **greater risk** associated. This increased risk could be due to uncertainty of outcome/chances of success (e.g. the evidence for the survival rate for a translocated population over the next 5 years is very mixed), the importance of the target (e.g. the target species is globally threatened or the action is costing many thousands of dollars), or the existence of other sensitivities around the action or target (e.g. public concerns around lethal predator control or increasing populations of carnivores). It is likely there will be a relatively small number (<10) of decisions of this type in most moderate-sized grant applications and funders may have a role in helping applicants identify these.

***3. How much detail?***

The level of detail of evidence needed for each decision will also depend on these same factors. A planned action to create nesting platforms at a lake to encourage breeding ospreys may require a few sentences to describe the relevant evidence, summarising previous studies on platform usage and breeding success. In comparison, a costly proposal to reintroduce a large herbivore population to a new site is likely to warrant a detailed description of the status and/or population trend of the species (e.g. as documented on the IUCN Red List), the suitability of the habitat, and the likely success of the proposed translocation (including the source and number of animals, the method of translocation and the release approach).

In such examples it may be pertinent to include evidence about the context, importance and feasibility of a proposed action (see Table 1 for an example). This could include information about acceptability, cost, logistical practicality, and the availability of necessary equipment and expertise. Again, this is likely to have the greatest benefits when presenting decisions which have substantial uncertainty associated.

**Table 1.** Example text describing evidence for the context and importance of an intervention

| **Evidence to support reintroduction of a new population of free-living bison to forests in southern Romania** |
| --- |
| *Bison were hunted to extinction in Romania in the 18th century (IUCN Bison Action Plan), but captive-bred populations are now successfully used for reintroductions in many parts of their previous range, providing numerous demonstrated environmental and societal benefits from improved ecosystem function and nature-based tourism (European Commission 2020). There were 6,200 free-living European bison in 2019 with the vast majority in Poland, Belarus and Russia. Only three small populations are present in Romania, all established since 2014 using translocations and soft releases of captive-bred individuals (Ministry of Forest and the Environment, Romania 2019). The aim is to eventually allow a large self-sustaining metapopulation of bison to become established in the region, with the potential for at least 1,100 individuals by 2050, as recommended by both IUCN (IUCN SSC Bison Specialist Group 2020) and the adopted National Red List plans (2018).* |

***4. How to present evidence***

It is initially likely to be most feasible for funders to ask applicants to describe their evidence use within a specific section of their application forms. However, over time we expect evidence to underpin aspects of the entire proposal.

Depending on the level of evidence needed (see section 3, above), the evidence supporting a proposed action can either be presented in a simple narrative paragraph or a more detailed tabular format. In either case, the aim is to provide sufficient information that the source, quality, relevance and extent of the evidence, and how it has been used in the decision-making process, is clear.

Here we give a series of illustrative examples of how evidence could be embedded in a project proposal, covering the narrative presentation of different types of evidence (Table 2a) and evidence of different levels of reliability and relevance (Table 2b), and a tabular example of a full evidence check (Table 3, based on Salafsky et al. 2019^[[1]](#footnote-1)^). The latter is appropriate if a decision has a higher risk associated and a detailed analysis of the evidence is required.

In some circumstances, applicants may not have the time, access or capacity to carry out full evidence searches as part of their application. In these cases, it may be more appropriate for applicants to describe their approach to evidence checking and for the funder to provide some support for this process (in terms of resources or expertise) as part of the funded project. Two possible situations and example texts are provided in Table 4.

**Table 2a**. Example text describing evidence from different sources

| **Source** | **Guidance** | **Examples** |
| --- | --- | --- |
| Scientific review or synthesis | Describe the number of studies and direction of evidence and cite the source of the review | *Thirteen of the 17 papers found on Conservation Evidence looking at the effect of the action ‘Designate a Marine Protected Area and prohibit all types of fishing’ showed a positive impact on crustacean abundance.*  Or  *Conservation Evidence only lists two papers on this action. Both were from very different species and were therefore not considered relevant.* |
| One or a small number of individual scientific studies | Provide the original source and describe the findings | *Rochefort & Bastien (1998) found that irrigation increased the number of Sphagnum moss shoots present after sowing in a replicated controlled study.* |
| Knowledge and experience | Provide sufficient details to assess the strength of evidence e.g. the source and basis of the information. | *Mary Smith, who has run the farm for thirty years, has observed that areas of overgrazing often become established with soft rush. Three other local farmers confirmed this was also seen on their land.* |

**Table 2b.** Example text describing evidence of different strengths

| **Quality and relevance** | **Example** |
| --- | --- |
| High quality and high relevance evidence | *There is good evidence to support the proposal to rewet peat to increase the Sphagnum cover. Of the 14 studies found on Conservation Evidence, 12 showed an increase in Sphagnum (one of the exceptions was a forested fen).* |
| Some evidence with moderate quality and varying relevance | *We propose building barrier fencing and underpasses along the stretch of road beside the reserve to reduce collisions and enable movement of moose. Evidence suggests barrier fencing is effective in reducing vehicle collisions across a wide range of mammals (51 out of 52 studies on Conservation Evidence). However, although in general underpasses are used by a wide range of mammals, moose were only twice seen to use underpasses in one study (Clevenger & Waltho 2000) and a study of radio-tracked moose found they did not use underpasses (Olsson & Widén 2008). Therefore, as well as using barrier fencing we plan to test two new designs of underpass: one with a wider entrance and one with planted trees, both of which may enhance use by moose (based on advice from local wildlife ranger).  We will monitor the number of moose using each type of underpass and also crossing the highway.* |
| Some evidence with mixed relevance | *We know of one study that has looked at the effect of a voluntary no-disturbance area on waterfowl: a before-and-after trial in the USA (Kenow et al. 2003) that found lower disturbance of waterfowl after the voluntary waterfowl avoidance area was created. Although our site is in the UK and the user groups differ from this study, interviews with key stakeholders have suggested there is support for such a scheme being effectively implemented.* |
| No relevant evidence | *No studies were found from Conservation Evidence or a search of the scientific literature describing the effectiveness of alternative means of obtaining seed mussels rather than dredging. However, there is extensive evidence on the overall damaging effects of dredging. We therefore propose to use seed mussels grown on suspended artificial substrates. Our trials over the last two years have shown these to be viable (data available on request) and we will collect and share data on the effectiveness of this option.* |

**Table 3.** Example of tabular presentation of evidence for a decision to introduce natural grazing with ponies to the montado habitat in Iberia

| **Evidence source^a^** | **Type of evidence^b^** | **Direction and strength of results^c^** | **Relevance (L/M/H)^d^** | **Quality (L/M/H)^e^*** |
| --- | --- | --- | --- | --- |
| Scientific study (Rolo & Moreno 2019) | Experimental and modelled | Ungrazed plots had longer droughts (up to 48 days longer) and took up 30% more water than grazed plots | Medium | Medium |
| Scientific study (Leal et al. 2019) | Experimental | A replicated controlled study found that moderate levels of grazing benefited most species of bird. | Medium | High |
| Rewilding Europe Grazelife report  www.grazelife.com | Observational? (not specified) | Grazed areas suffer less from large-scale wildfires than surrounding areas that are abandoned (shrub encroachment) | High | Low |
| https://www.conservationevidence.com/actions/1628 | Synthesis | Three of five studies across Europe found that increasing grazing intensity increased plant diversity in shrublands | Low | Medium |

^a^ e.g. Peer-reviewed article, expert opinion, grey literature report, personal experience

^b^ e.g. Synthesis, experimental, observational, anecdotal, theoretical/modelling

^c^ Was the result strongly positive, weakly positive, mixed, or no effect?

^d^ How relevant is the evidence in terms of geography, taxa or habitat? In addition, does the evidence relate directly to the intervention effectiveness?

^e^ Depends on the type of evidence, but also sample size & experimental design

**Table 4.** Example text describing applicants’ approach for evidence checking

| **Context** | **Description** | **Example text** |
| --- | --- | --- |
| Evidence available but review and assessment delayed until grants is awarded | Appropriate where the assessment of the evidence for a decision will not affect the overall feasibility of the proposal, but the details of how it is to be implemented will be important in determining success. | *There is substantial evidence on meadow creation, including 36 actions reviewed on Conservation Evidence and considerable expertise at the local agricultural college. Prior to carrying out the grassland restoration, we will review this literature and consult widely at the local and regional level to determine the most effective methodology.* |
| Providing assurance that future decisions will be evidence based | Applicable in situations where substantial decision making takes place after the grant is awarded. Here, the aim is to demonstrate that evidence review is already embedded into organisational processes. | *The precise conservation actions to be implemented are not yet known, as they will depend on the land acquired and results of stakeholder consultation. Since 2020 we have become an evidence-led organisation, employing an Evidence and Implementation Manager who oversees the use and creation of evidence. This includes establishing a new test of an action each year and organising an annual meeting of regional wetland reserve managers to share experiences and lessons learned.* *Before undertaking any actions we will undertake an evidence review and expert consultation to inform decision making.* |

***5. Guidance for funders on assessing evidence use in applications***

When assessing the evidence base provided by applicants, it is important to remain aware that reviewing evidence may be a new process for applicants and it is likely to be most useful if the process is supportive and collaborative rather than a strict judgement of the evidence base. It is particularly important to ensure the process is fair to projects working on species, ecosystems or regions where evidence is scarce.

The aim of asking applicants to describe the evidence base for their proposal is to aid the applicant, encourage transparent decision making, and improve the effectiveness of the final project. Therefore, it may or may not be appropriate for evidence use to be a criterion on the assessors’ score sheet; the main aspiration is to ensure that applicants have gone through a rigorous process as part of their decision making, even if the formal scientific evidence is sparse.

If applicants are able to demonstrate that they have considered the evidence base behind key decisions in planning the objectives and implementation of their proposal, this will provide assurance of transparent and carefully thought out planning processes, increasing the chances of delivering a successful conservation project.

1. See Salafsky et al. (2019) Defining and using evidence in conservation practice. *Conservation Science and Practice,* 1(5), e27 for a more detailed framework for evidence use [↑](#footnote-ref-1)
